# Sodium Tanshinone IIA Sulfonate Ameliorates Bladder Fibrosis in a Rat Model of Partial Bladder Outlet Obstruction by Inhibiting the TGF-β/Smad Pathway Activation

**DOI:** 10.1371/journal.pone.0129655

**Published:** 2015-06-10

**Authors:** Xiaoxiao Jiang, Yaping Chen, Haitao Zhu, Bo Wang, Ping Qu, Renfu Chen, Xiaoqing Sun

**Affiliations:** 1 Xuzhou Medical College, Xuzhou, Jiangsu, China; 2 Department of Urologic Surgery, the Affiliated Hospital of Xuzhou Medical College, Xuzhou, Jiangsu, China; 3 Department of Urologic Laboratory, Xuzhou Medical Affiliated Hospital, Xuzhou, Jiangsu, China; 4 Department of Urologic Surgery, Yancheng City No.1 People’s Hospital, Yancheng, Jiangsu, China; 5 Department of Oncology, Yancheng City No.1 People’s Hospital, Yancheng, Jiangsu, China; University of Louisville, UNITED STATES

## Abstract

Transforming growth factor (TGF)-β1 is known to play a pivotal role in a diverse range of biological systems including modulation of fibrosis in several organs. The precise role of TGF-β/Smad signaling in the progression of bladder fibrosis secondary to partial bladder outlet obstruction (PBOO) is yet to be conclusively. Using a rat PBOO model, we investigated TGF-β1 expression and exaimined whether sodium tanshinone IIA sulfonate (STS) could inhibit TGF-β/Smad signaling pathway activation and ameliorate bladder fibrosis. Forty-eight female Sprague-Dawley rats were randomly divided into three groups: sham operation group (n = 16), PBOO operation without STS treatment group (n = 16) and PBOO operation with STS treatment group (n = 16). Thirty-two rats underwent the operative procedure to create PBOO and subsequently received intraperitoneal injections of STS (10 mg/kg/d; n = 16) or vehicle (n = 16) two days after the surgery. Sham surgery was conducted on 16 rats, which received intraperitoneal vehicle injection two days later. In each of the three groups, an equal number of rats were sacrificed at weeks 4 and 8 after the PBOO or sham operation. The TGF-β/Smad signaling pathway was analyzed using western blotting, immunohistochemical staining and reverse transcriptase polymerase chain reaction (RT-PCR). One-way analysis of variance was conducted to draw statistical inferences. At 4 and 8 weeks, the expression of TGF-β1 and phosphorylated Smad2 and Smad3 in STS-treated PBOO rats was significantly lower than in the PBOO rats not treated with STS. Alpha smooth muscle actin (α-SMA), collagen I and collagen III expression at 4 and 8 weeks post PBOO was lower in STS-treated PBOO rats when compared to that in PBOO rats not treated with STS. Our findings indicate that STS ameliorates bladder fibrosis by inhibiting TGF-β/Smad signaling pathway activation, and may prove to be a potential therapeutic measure for preventing bladder fibrosis secondary to PBOO operation.

## Introduction

Partial urinary bladder outlet obstruction (PBOO) is frequently observed in various clinical diseases such as benign prostate hyperplasia (BPH), neurogenic bladder, bladder neck stricture or posterior urethral stenosis. Bladder function is altered following bladder outlet obstruction and cannot be restored entirely, even after the obstruction is relieved. The characteristics of PBOO include bladder smooth muscle hyperplasia, increased strength of detrusor contractility, or deposition of extracellular matrix (ECM)[1]. Bladder tissue fibrosis is an inevitable consequence of excessive ECM accumulation that occurs virtually in all cases of chronic bladder outlet obstruction. The development of bladder wall tissue fibrosis in the setting of partial outlet obstruction is thought to be due to increased levels of nerve growth factor, basic fibroblast growth factor, connective tissue growth factor or transforming growth factorβ1(TGF-β1)[2–5].

Among the diverse regulatory factors involved, TGF-β1 might play a critical role in bladder fibrosis. Transforming growth factor-β1, a dimmer, composed of two equal subunits containing 112 amino acids, is a multifunctional growth factor. It initiates intracellular responses by binding to the specific transmembrane receptors which have intracellular serine/threonine kinase activity[6]. These activated receptors further lead to phosphorylation of receptor-associated Smad2/3 proteins, which are capable of combining with Smad4. Finally, this complex of activated receptors and proteins regulates the transcription of target genes. A study using a 6-week bladder outlet obstruction model, demonstrated increase in the the weight of bladder tissue and decrease in that the detrusor contractile force, which was accompanied by a significant increase in urine TGF-β1 [7]. The study reported a negative correlation between urine TGF-β1 levels and detrusor contractile force. In a study conducted by Anumanthan et al., bladder compliance was found to be increased in partially obstructed TGF-β type II receptor knockout mice, with decreased collagen deposition[8]. Blockage of the TGF-β/Smad signaling pathway by using various strategies such as anti-transforming growth factor (TGF)-β1 antibody, tanshinone II A, microRNAs and small interfering RNA have provided vital evidence pointing towards the crucial role of the pathway in development of fibrosis in various tissues and organs [9–12].

Sodium tanshinone IIA sulfonate (STS) is a water-soluble compound extracted from the root of a Chinese herbal Salvia *miltiorrhiza* Bunge. It has been widely used in traditional Chinese medicine for treating cardiovascular diseases, inflammatory disorders, and hepatitis [10, 13, 14]. While accumulated evidence shows that STS inhibits tissue or organ fibrosis, the definite mechanism by which this occurs is a major topic of discussion. Various studies have demonstrated that STS ameliorates cardiac, renal and atrial fibrosis by TGF-β1 signaling [10, 15, 16]. TGF-β1 is also thought to play a pivotal role in the deterioration of the bladder tissue fibrosis that follows PBOO. The present study was conducted to assess whether STS prevents the development of bladder fibrosis by inhibiting the TGF-β/Smad signaling pathway in a rat PBOO model.

## Materials and Methods

### Animals and animal models of partial bladder outlet obstruction (PBOO)

Adult female Sprague Dawley rats weighing 160-200g were obtained from the Experimental Animal Centre of the Xuzhou Medical College (Xuzhou, China). This study was approved by Xuzhou Medical College Committee for Animal Experimentation. All animal experiments were performed in accordance with the guidelines set by Xuzhou Medical College Committee for Animal Experimentation (Permit Number: S2010-0011). The study was carried out in strict accordance with the recommendations in the Guide for the Care and Use of Laboratory Animals of the National Institutes of Health. All animals were housed in a temperature controlled room, maintained between 21°C and 25°C, exposed to a 12 hour light–dark cycle, and had free access to food and water.

Forty eight adult female Sprague-Dawley rats were randomly divided into three groups: sham operation group (n = 16), PBOO operation without STS treatment group (n = 16) and PBOO operation with STS treatment group (n = 16). In our study, the PBOO procedure performed was a slightly modified version of the technique described previously[17]. The animals were anaesthetized using intraperitoneal injection of sodium pentobarbital (Sigma, 40 mg/kg). After disinfection with iodine tincture, a catheter of 1mm diameter was inserted into the urethra, A midline incision was made on the abdominal to expose the urethrovesical junction. The proximal urethra was tied gently but snugly around the urethrovesical junction with a 3–0 silk thread, the incision was closed, and the catheter was removed to produce PBOO. Gentamicin (0.1 mg/kg) was administered intraperitoneally. The same procedure was conducted on the rats in the sham group, but without applying the ligature around the proximal urethra. The rats in the sham operation group and PBOO operation without STS treatment group were injected intraperitoneally with sterile normal saline two days post operation, while the rats in PBOO operation with STS treatment group received intraperitoneal injection of STS (10mg/kg/d, Shanghai NO.1 Biochemical& Pharmaceutical Co., Ltd, China)[18]. All drugs were dissolved in normal saline and injected intraperitoneally. Half of the rats in each of the three groups were sacrificed in four weeks after the procedure, while the remaining half were sacrificed eight weeks post-procedure the remaining. After the sacrifice, part of the bladder tissues were immediately harvested for histology or immunochemistry, and the remaining tissues were stored in liquid nitrogen for performing in RT-PCR or western blotting.

### Histological and immunohistochemical measurements

Bladder tissues fixed in 4% buffered paraformaldehyde solution were embedded in paraffin and cut into 4μm sections. The sections were stained with Masson’s trichrome staining kit (KeyGen Biotech, Nanjin, China) and hematoxylin and eosin staining for histological analysis and assessment of interstitial collagen content. Immunohistochemical assays were performed by using anti-TGFβ1 (Santa Cruz Technology, USA), anti-phospho-Smad2 (Bioworld Technology, USA), anti-phospho-Smad3 (Cell signaling), anti-collagen-I (Bioworld Technology, USA), anti-collagen III (Bioworld Technology, USA), and anti-a-SMA (Santa Cruz Technology, USA). The sections were dewaxed and quenched with 3% hydrogen peroxide for 10minutes to block the endogenous peroxidase activity, followed by serum-free protein blocking (ZSGB-BIO, Beijing, China). The sections were then incubated with various antibody mentioned above, followed by incubation with biotinylated anti-rat IgG and HRP-Streptavidin (ZSGB-BIO, Beijing, China). Diaminobenzidine was used as a substrate and counterstaining was performed with hematoxylin. A negative control was prepared by using an irrelevant IgG. Four fields were randomlyselected from each sample and examined under light microscopy (400x magnifiation) and the evaluator was blinded to the identity of the group of rats from which the sections were prepared to avoid an bias. The results of immunochemistry were analyzed by using an image analyze software (Image J, Institutes of Health).

### Western blotting

Western blotting was performed for the detection of TGFβ1, smad2/3, phosphorylated Smad2/3, a-SMA, collagen I and collagen III. Total proteins were extracted from bladder tissues and analyzed with bicinchoninic acid protein concentration assay kit (Beyotime Institutes of Biotechnology, Shanghai, China). Proteins were isolated from the samples by SDS-PAGE electrophoresis and then transferred onto nitrocellulose membrane. After blocking with 5% nonfat milk or 2% bovine serum albumin (BSA), the membranes were incubated at 4°C overnight with a rabbit anti-rat polyclonal TGFβ1 (Santa Cruz Technology, 1:800dilution), smad2/3 antibody (Santa Cruz Technology, 1:1000dilution), p-smad2 antibody (Bioword Technology, 1:800dilution), p-smad3 (Cell signaling,1:1000dilution), α-SMA(Cell signaling,1:1000dilution), collagen I (Bioworld Technology, 1:800dilution) and collagen III (Bioworld Technology,1:800dilution). The second antibody was a goat anti-rabbit IgG (1:10000dilution, Li-COR, USA), which was diluted in TBST. Signals were scanned and visualized by Odyssey Infrared Imaging System (LiCor Inc., Lincoln, NE). Density of western strap was quantified by Image J software, with the gray value representing the amount of expressed target protein. Relative protein levels were calculated by comparing the gray value of target protein to the amount of β-actin protein.

### Real-time reverse transcriptase polymerase chain reaction

Total RNA was extracted from the frozen bladder tissues using Trizol Reagent(Tiangen Biotech Co.,Ltd. Beijing, China) in accordance with the vendor’s instructions. First strand cDNA was synthesized using Oligo(dT)_15_ primers in a first strand synthesis kit (Promega Tech, Co., Ltd, USA). Real-time PCR was conducted using PCR Master Mix (Promega Tech, Co., Ltd, USA) according to the vendor’s recommendations. Total RNA was reverse-transcribed and subjected to PCR as follows: 95°C for 2minutes followed by 30 cycles, each cycle comprising of 95°C for 30s, 56°C for 30s and 72°C for 45s,and a final extension at 72°C for 7minutes.The primer sequences amplified were as follows.

- Rat TGFβ1: sense: 5’-CCGCAACAACGCAATCTA-3’, anti-sense: 5’-TGAGGAGCAGGAAGGGTC -3’
- Smad2: sense: 5’-CCACTACCAGAGGGTGGAGA-3’, anti-sense: 5’-TAACTG GCTGCAATCCAAG-3’
- α-SMA: sense: 5’-CTGAGCGTGGCTATTCCTTC-3’, anti-sense: 5’-CGTCATACTCCTGTTTGCTGA-3’;
- Smad3: sense 5’-AACTGCAGT GCCGCTATCC -3’, anti-sense: 5’- CCAGCGGGGAAGTTAGTGTT-3’
- Collagen1: sense 5’-ATCAGCCCAAACCCCAAGGAGA-3’, anti-sense: 5’-CGCAGGAAGGT CAGCTGGATAG-3’

Collagen 3: sense 5’-TGATGGGATCCAATGAGGGAGA-3’, anti-sense: 5’-GAG TCT CATGGCCTTGCGTGTTT-3’

Primer sequences for glyceraldehyde 3-phosphate dehydrogenase (GAPDH) were 5’-CGTCATTGACCTCAACTACATG-3’ (sense) and 5’-CTTCTCC ACGGTGGTGAAGAC-3’ (anti-sense) as an internal standard. Reverse-transcribed PCR products were detected by 2% agarose gel electrophoresis. Expression levels of target mRNA were represented by ratios of TGFβ1/ GAPDH, Smad2/ GAPDH, Smad3/ GAPDH, α-SMA/ GAPDH, Collagen1/ GAPDH, Collagen3/ GAPDH mRNA and showed as the mean ±S.D.

### Statistic analysis

The results were presented as the mean±S.D. A statistic comparison of differences between groups was performed using the one-way analysis of variance by the SPSS software, vesion13.0 for Windows. A *P* <0.05 was regarded as statistically significance.

## Results

### Effects of STS on bladder tissue histology in rat PBOO model

**Fig 1:**
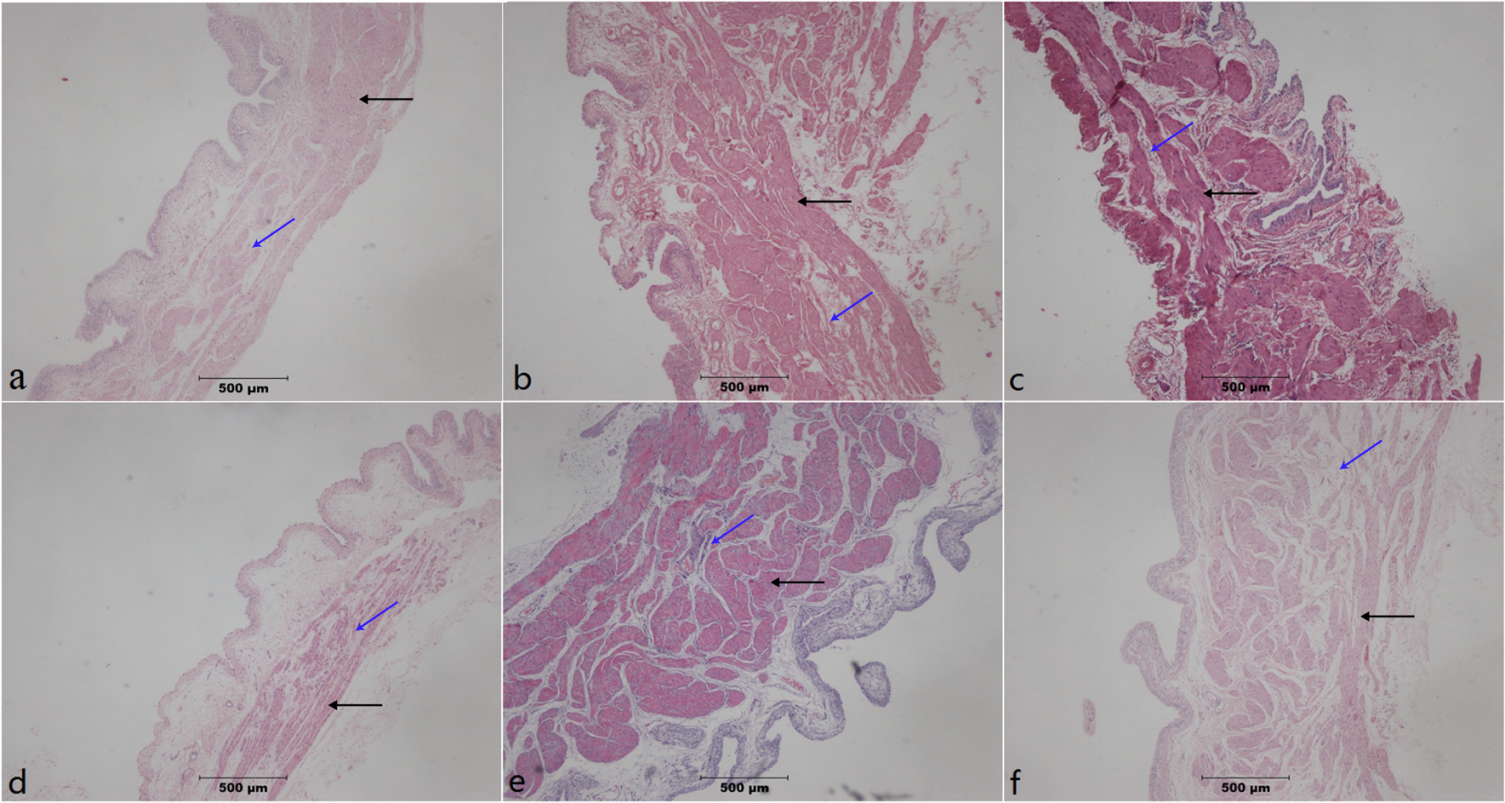
H&E-stained paraffin-embedded rat bladder sections. In PBOO rats, significant detrusor muscle hypertrophy, alongside a progressive increase in fibrosis (dyed pale pink, blue arrow) and loss of normal muscle tissue (dyed purple or red, black arrow)architecture was observable at 4 and 8 weeks of PBOO. Fibrosis was inhibited in the STS group at (a) week 4 in Sham group, (b) week 4 after PBOO, (c) week 4 after PBOO+STS treatment, (d) week 8 after Sham group, (e) week 8 after PBOO, and(f) week 8 after PBOO+STS treatment. The PBOO and STS groups show detrusor hypertrophy and collagen deposition. Original magnifications, ×40.

Paraffin-embedded sections from PBOO operation without STS treatment group and PBOO operation with STS treatment groups were stained with hematoxylin and eosin and examined. The two groups of rats which had undergone PBOO procedure exhibited significant bladder detrusor hypertrophy at weeks 4 and 8. Furthermore, a progressive increase in fibrosis and loss of normal tissue architecture from week 4 to 8 was observed in the group which had not been administered STS. However, fibrosis was inhibited in the group which had been treated with STS (Fig 1).

### STS attenuates progressive bladder fibrosis by suppressing the expression of excess extracellular matrix proteins

**Fig 2:**
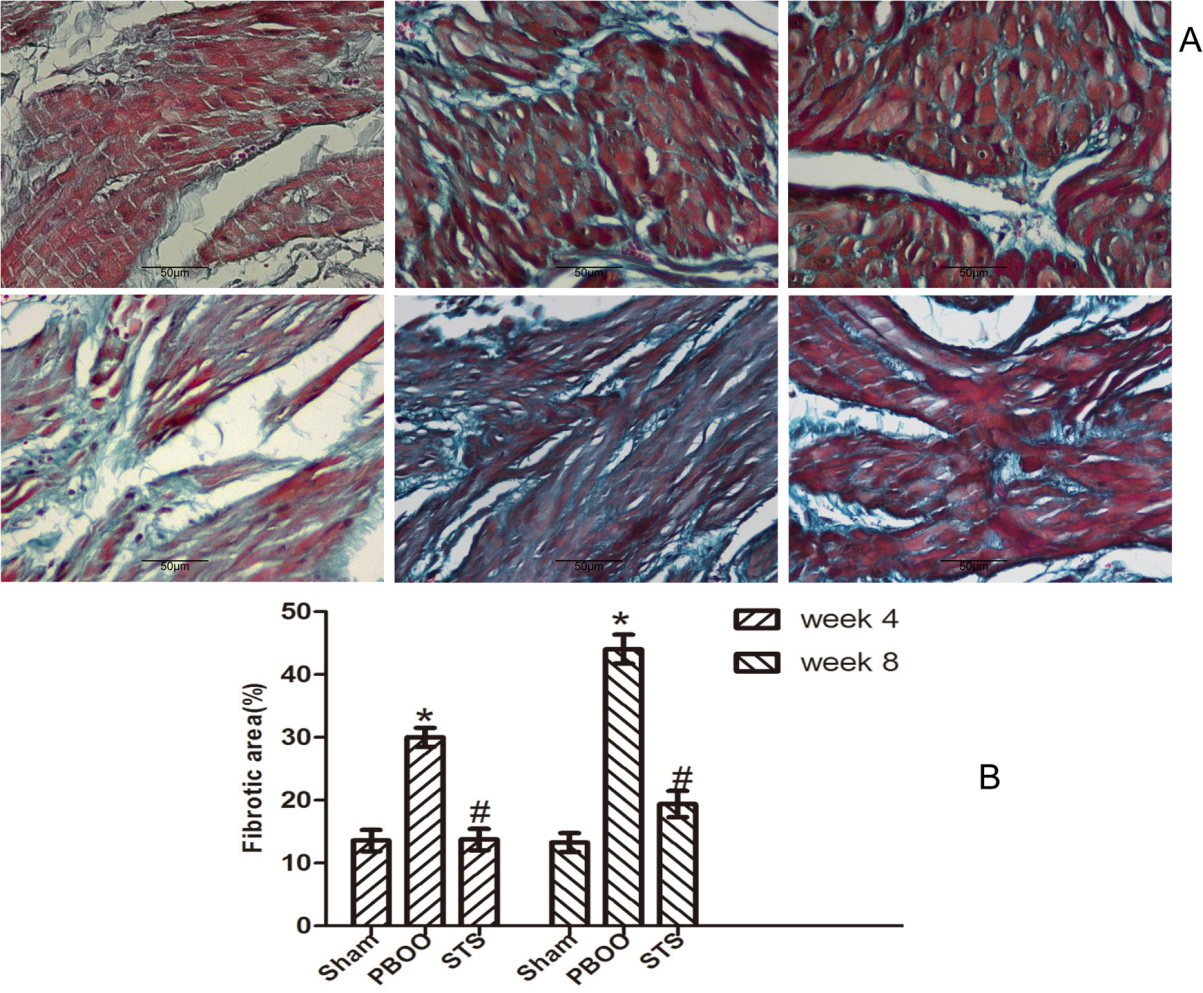
Masson’s trichrome-stained sections show that PBOO induces bladder fibrosis and STS suppresses bladder fibrosis. Collagen fibril is represented by the green staining and muscle is purple (A) Representative Masson’s trichrome–stained sections. (a) Sham group at 4 weeks, (b) week 4 after PBOO, (c) week 4 after PBOO+STS treatment, (d) week 8 after Sham group, (e) 8 weeks after PBOO, (f) 8 weeks after PBOO+STS treatment. (B) Fibrotic area scores of the bladder tissues. Each bar represents the mean±SD of eight rats. *P<0.05 *vs*. Sham group (n = 8); ^#^ *P*<0.05 *vs* PBOO group. Original magnifications, ×400.

**Fig 3:**
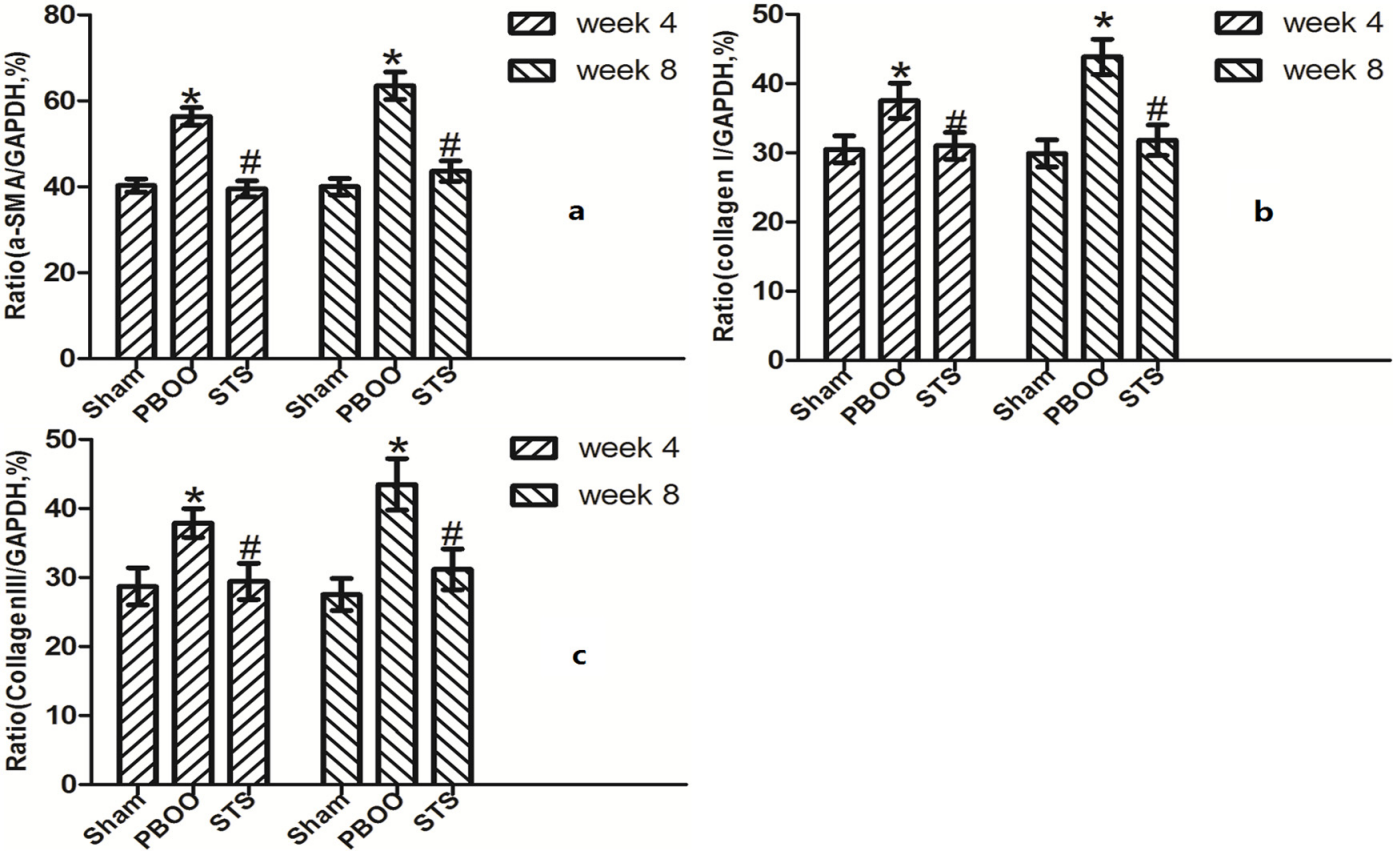
RT- PCR of a-SMA (*Acta2*), collagen I (*Col1a1*), collagen III (*Col3a1*) mRNA expression *in vivo* after STS treatment in the PBOO model. (a) *Acta2* mRNA, (b) *Col1a1* mRNA, (c) *Col3a1* mRNA. *Acta2*, *Col1a1* and *Col3a1* mRNA expression was significantly higher in PBOO rats at weeks 4 and 8 compared with Sham rats (*P*<0.05, n = 8). STS treatment led to decreased *Acta2*, *Col1a1*, *Col3a1* mRNA expression (*P*<0.05, n = 8). Each bar represents the mean±SD for eight rats. *P<0.05 *vs*. sham group (n = 8); ^#^ *P*<0.05 *vs*. PBOO group.

**Fig 4:**
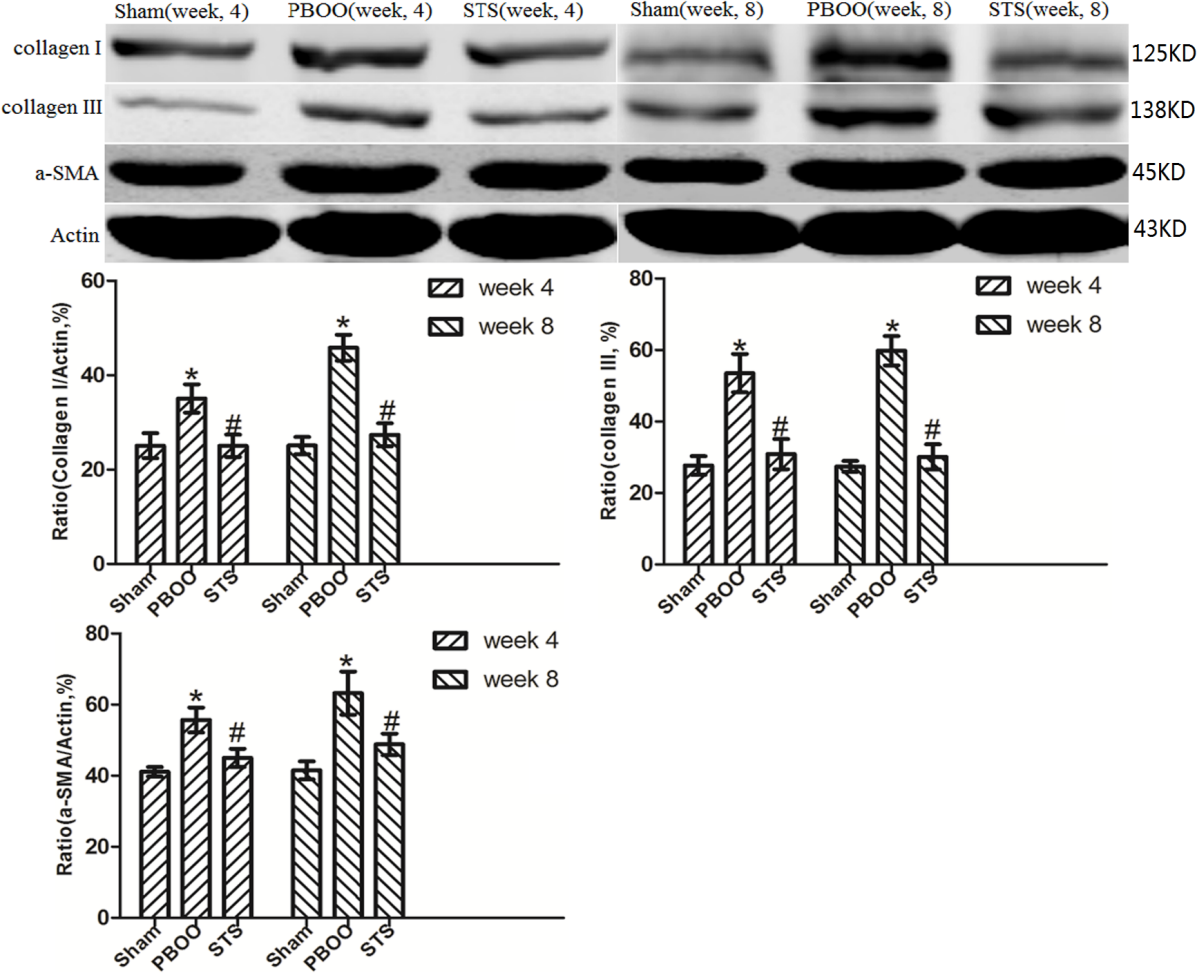
Western blotting analyses of a-SMA, collagen I, and collagen III protein expression in vivo after STS treatment in the PBOO model. a-SMA, collagen I and collagen III expression was significantly higher in PBOO rats at weeks 4 and 8 compared with the Sham group (*P*<0.05, n = 8). Each bar represents the mean±SD of 8 rats. *P<0.05 *vs*. sham group (n = 8); ^#^ *P*<0.05 *vs*. PBOO group.

**Fig 5:**
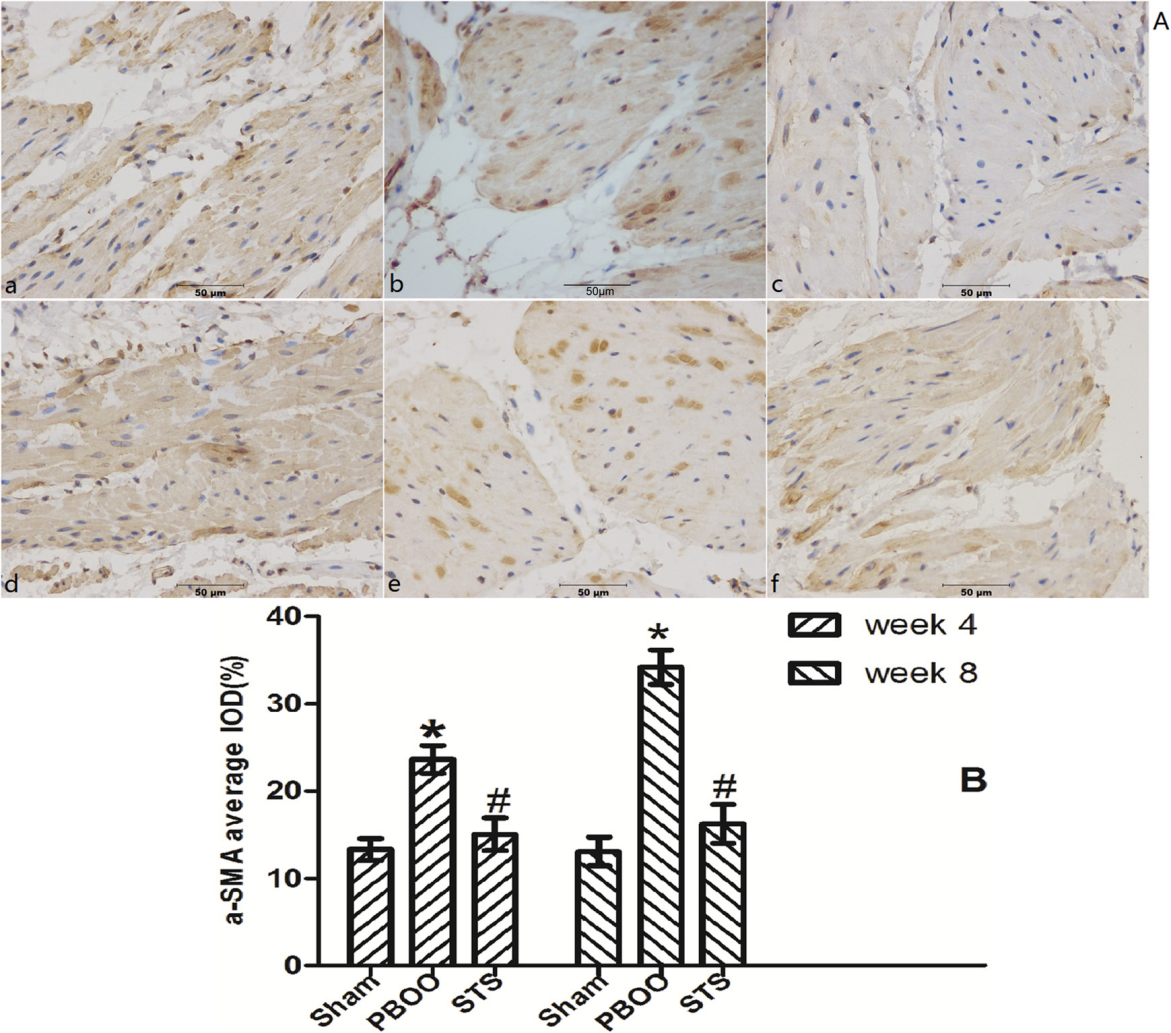
STS treatment suppresses a-SMA expression in the PBOO model. (A) Representative a-SMA-immunostained rat bladder sections. (a) Sham group at 4 weeks, (b) week 4 after PBOO, (c) week 4 after PBOO+STS treatment, (d) week 8 after Sham group, (e) week 8 after PBOO, (f) week 8 after PBOO+STS treatment. a-SMA expression was significantly higher in PBOO rats at weeks 4 and 8 compared with Sham groups (*P*<0.05, n = 8). STS treatment induced decreased a- SMA expression (*P*<0.05, n = 8). (B) Densitometric analyses performed from 8 independent experiments. Each bar represents the mean±SD for 8 rats. *P<0.05 *vs*. sham group (n = 8); ^#^ *P*<0.05 *vs*. PBOO group. Original magnifications, ×400. IOD, integrated optical density.

**Fig 6:**
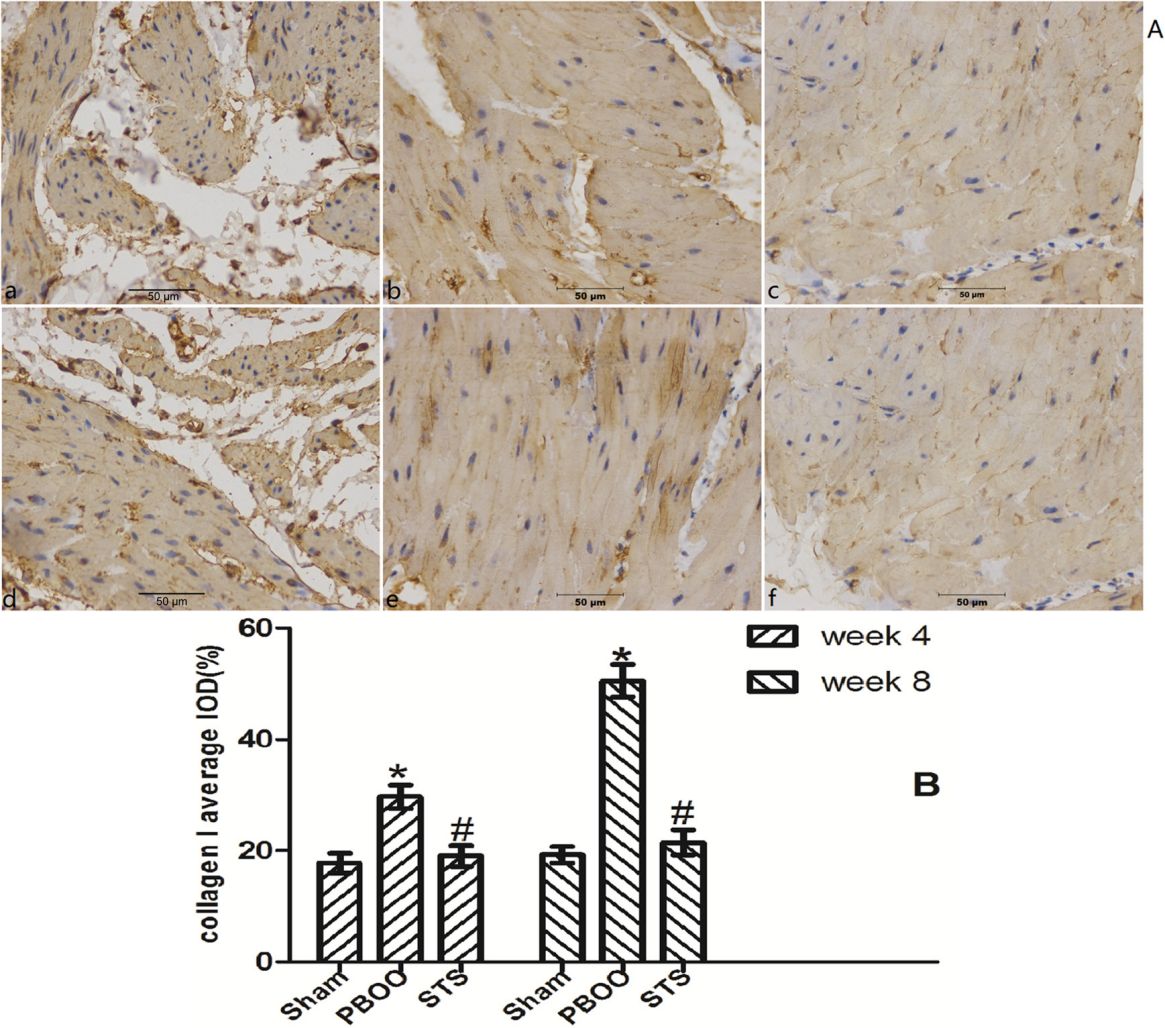
STS treatment suppresses collagen I expression in the PBOO model. (A) Representative collagen I-immunostained rat bladder sections. (a) Sham group at 4 weeks, (b) week 4 after PBOO, (c) week 4 after PBOO+STS treatment, (d) week 8 after Sham group, (e) week 8 after PBOO, (f) week 8 after PBOO+STS treatment. Collagen I expression was significantly higher in PBOO rats at weeks 4 and 8 compared with the Sham groups (*P*<0.05, n = 8). STS treatment induced decreased collagen I expression (*P*<0.05, n = 8). (B) Densitometric analyses were performed from 8 independent experiments. Each bar represents the mean±SD of 8 rats. *P<0.05 *vs*. Sham group (n = 8); ^#^ *P*<0.05 *vs*. PBOO group. Original magnifications, ×400.

**Fig 7:**
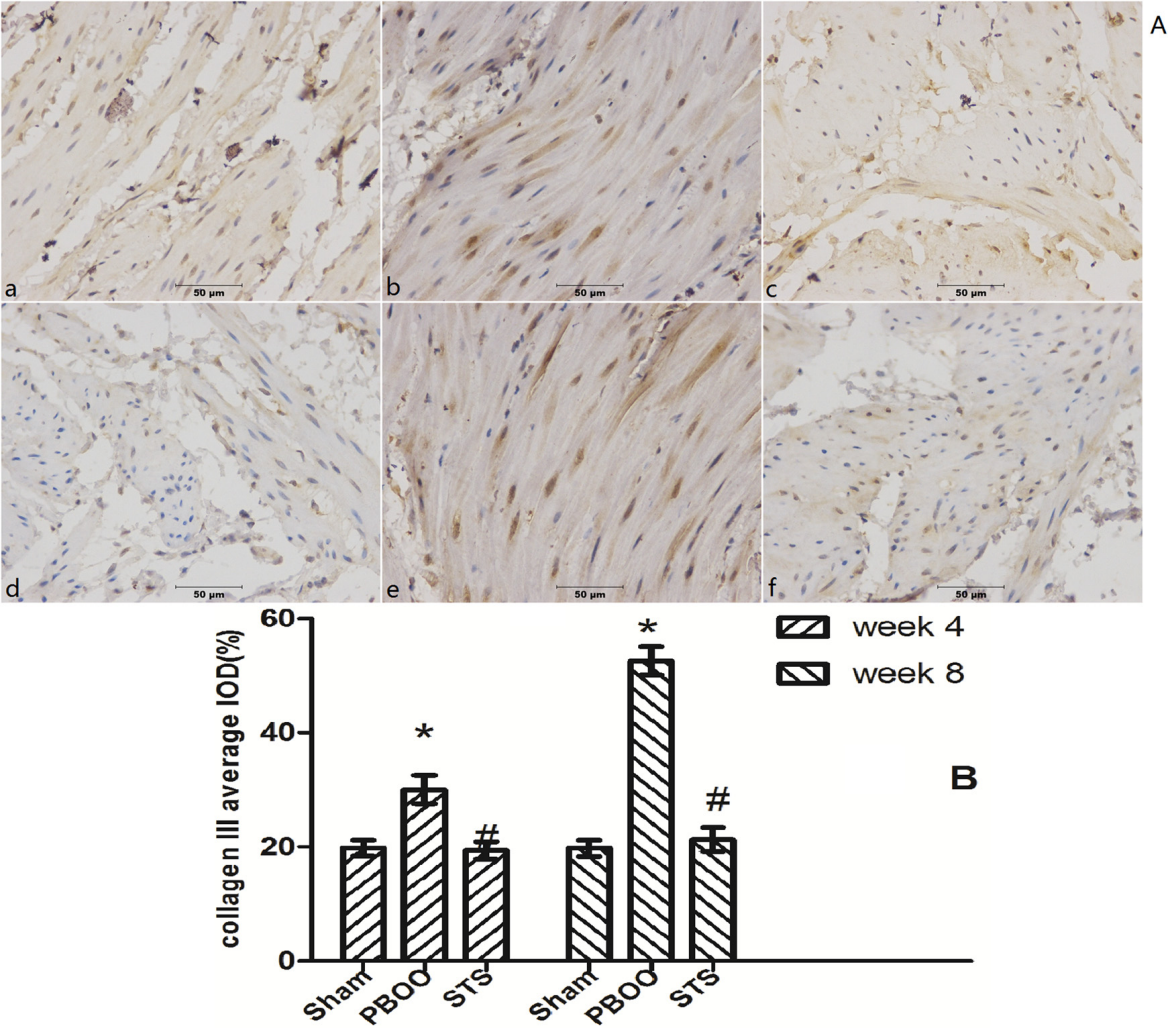
STS treatment suppresses collagen III expression in the PBOO model. (A) Representative collagen III immunostained rat bladder sections. (a) Sham group at 4 weeks, (b) week 4 after PBOO, (c) week 4 after PBOO+STS treatment, (d) week 8 after Sham group, (e) week 8 after PBOO, (f) week 8 after PBOO+STS treatment. Collagen III expression was significantly higher in the PBOO rats at weeks 4 and 8 compared with the Sham groups (*P*<0.05, n = 8). STS treatment induced decreased collagen III (*P*<0.05, n = 8). (B) Densitometric analyses performed from 8 independent experiments. Each bar represents the mean±SD of 8 rats. *P<0.05 *vs*. Sham group (n = 8); ^#^ *P*<0.05 *vs*. PBOO group. Original magnifications, ×400.

The inhibitory effects of STS on bladder fibrosis, the expression of α-SMA, collagen I and collagen III protein and their mRNA were consistently detected by western blotting, immunohistochemistry, and RT-PCR. Masson’s trichrome staining of bladder tissue sections showed the progressive increase in collagen deposition in the PBOO rats without STS treatment, however, significantly decreased in the PBOO rats with STS treatment (Fig 2). RT-PCR analysis showed that α-SMA, collagen I, and collagen III expression were decreased in PBOO rats with STS treatment rats at weeks 4 and 8 (*P*<0.05, n = 8; Fig 3). The western blot and immunohistochemistry findings were also consistent with that of the RT-PCR resultsmRNA expression (*P*<0.05, n = 8; Figs 4, 5, 6 and 7).

### Blockage of TGF-β/Smad activation was found to be a critical mechanism in STS prevention of bladder fibrosis in PBOO rats with STS treatment

**Fig 8:**
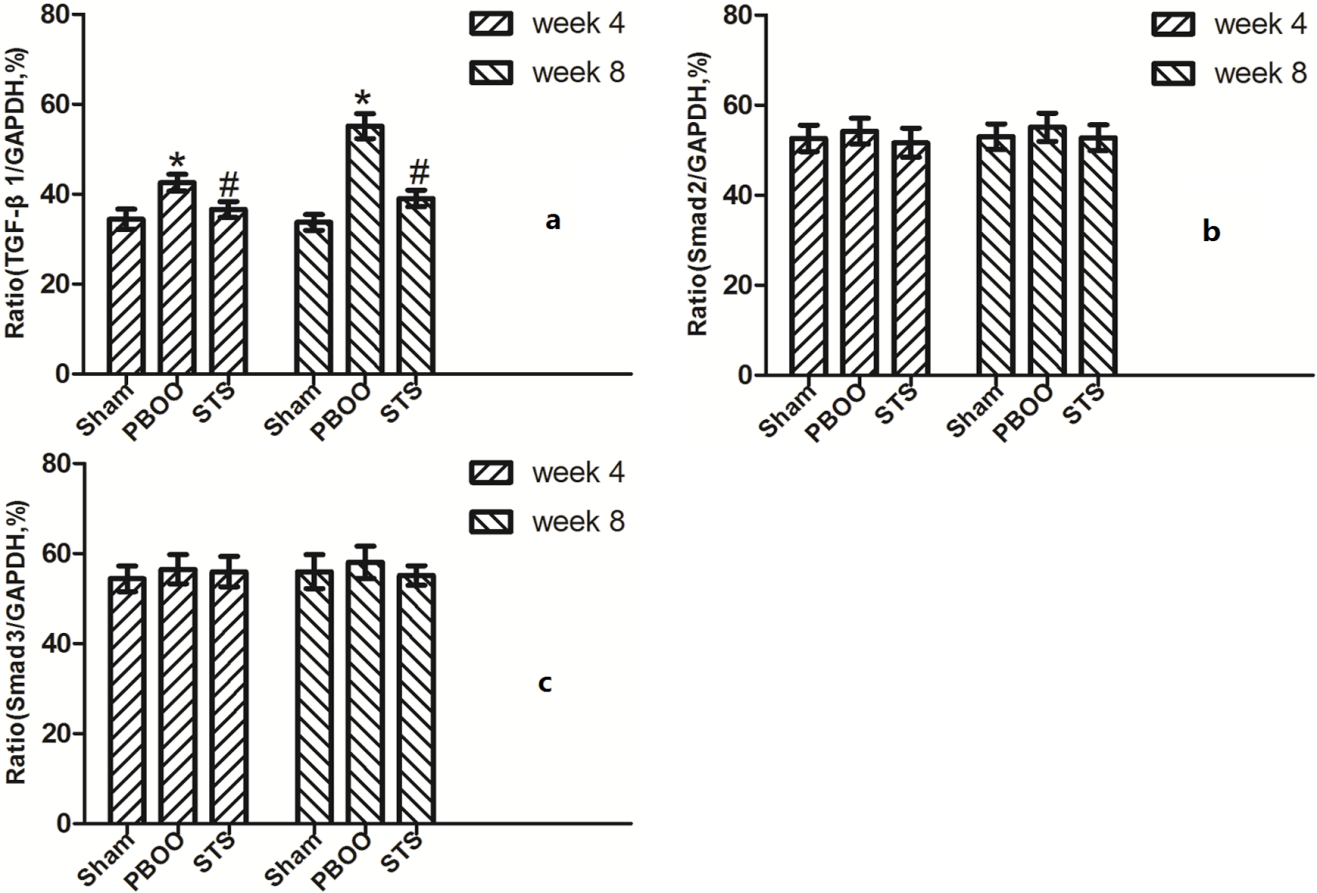
RT-PCR of (a)*Tgfb1*, (b)*Smad2* and (c)*Smad3* mRNA expression *in vivo* after STS treatment in PBOO model. Compared with the Sham group, *Tgfb1*, *Smad2* and *Smad3* mRNA expression in the PBOO group was not significantly different. Densitometric analyses were performed from 8 independent experiments. Each bar represents the mean±S.D of 8 rats. *P<0.05 *vs*. sham group (n = 8); ^#^ *P*<0.05 *vs*. PBOO group.

**Fig 9:**
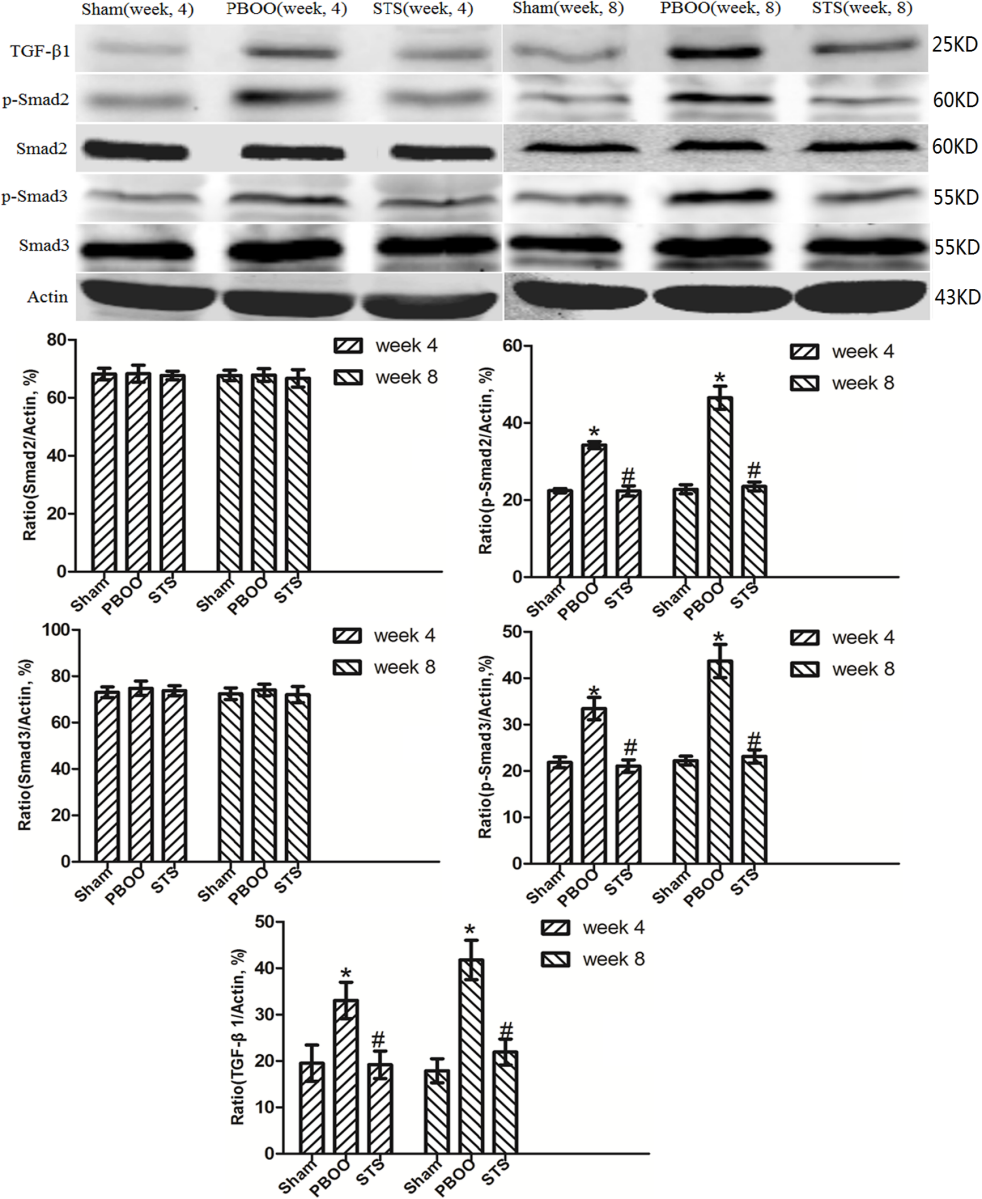
Western blotting analyses of TGF-β1, Smad2 and Smad3 protein expression *in vivo* after STS treatment in the PBOO model. Compared to the Sham group, TGF-β1 and phosphorylated Smad2/3 expression but not total Smad2/3 expression in PBOO was significantly higher at weeks 4 and 8 (*P*<0.05, n = 8). Densitometric analyses performed from 8 independent experiments. Each bar represents the mean±SD of 8 rats. *P<0.05 *vs*. sham group (n = 8); ^#^ *P*<0.05 *vs*. PBOO group.

**Fig 10:**
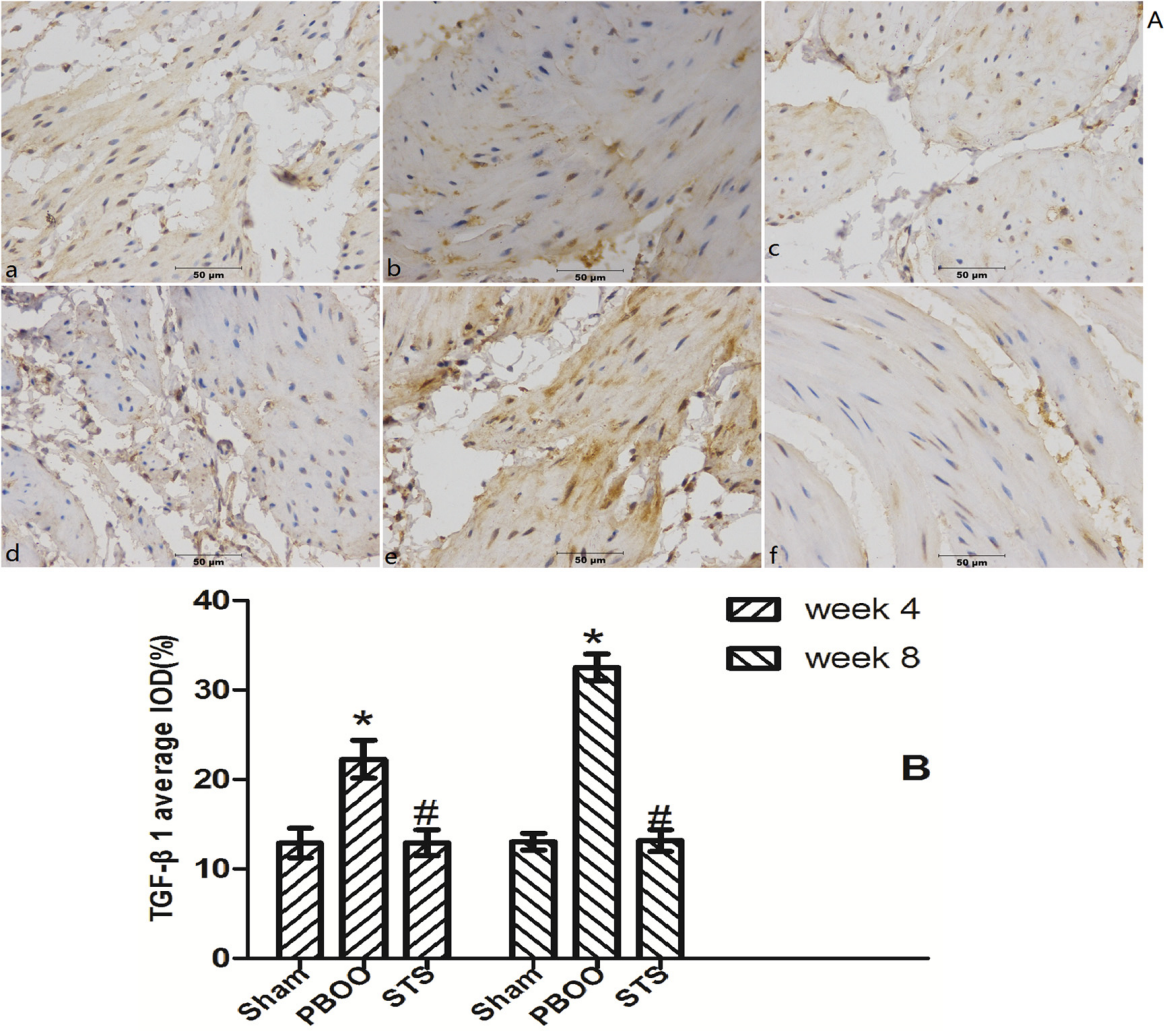
STS treatment suppresses TGF-β1 expression in the PBOO model. (A) Representative TGF-β1-immunostained rat bladder sections. (a) Sham group at 4 weeks, (b) week 4 after PBOO, (c) week 4 after PBOO+STS treatment, (d) week 8 after Sham group, (e) week 8 after PBOO, (f) week 8 after PBOO+STS treatment. TGF-β1 expression was significantly higher in the PBOO rats at weeks 4 and 8 compared with the Sham groups (*P*<0.05, n = 8). STS Treatment induced decreased TGF-β1 expression (*P*<0.05, n = 8). (B) Densitometric analyses performed from 8 independent experiments. Each bar represents the mean±SD for 8 rats. *P<0.05 *vs*. sham group (n = 8); ^#^ *P*<0.05 *vs*. PBOO group. Original magnifications, ×400.

**Fig 11:**
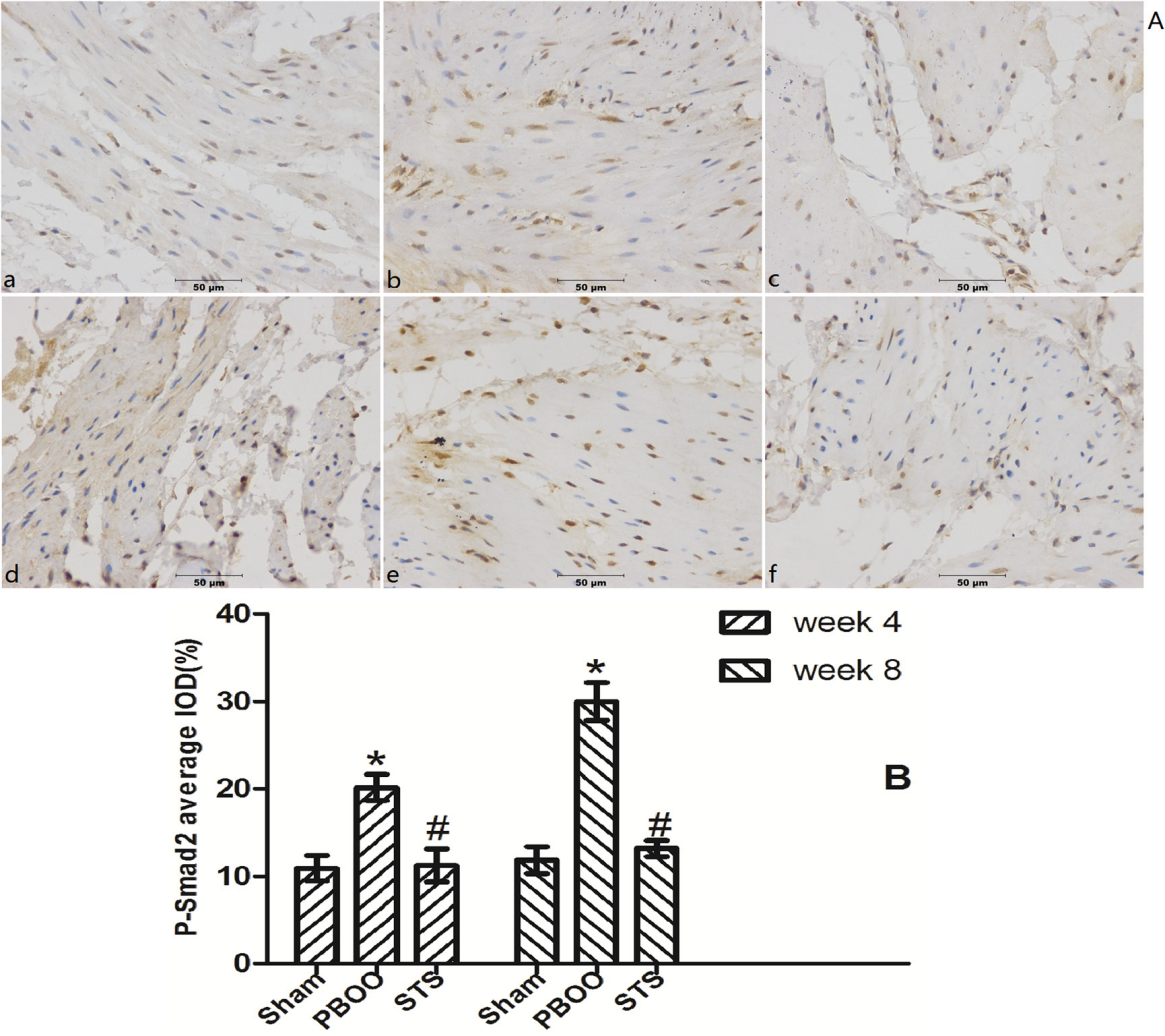
STS treatment suppresses expression of phosphorylated Smad2 in the PBOO model. (A) Representative Phosphorylated Smad2-immunostained rat bladder sections. (a) Sham group at 4 weeks, (b) week 4 after PBOO, (c) week 4 after PBOO+STS treatment, (d) week 8 after Sham group, (e) week 8 after PBOO, (f) week 8 after PBOO+STS treatment. Expression of Phosphorylated Smad2 was significantly higher in the PBOO rats at weeks 4 and 8 compared with the Sham groups (*P*<0.05, n = 8). STS Treatment induced decreased Phosphorylated Smad2 (*P*<0.05, n = 8). (B) Densitometric analyses performed from 8 independent experiments. Each bar represents the mean±SD for 8 rats. *P<0.05 *vs*. sham group (n = 8); ^#^ *P*<0.05 *vs*. PBOO group. Original magnifications, ×400. IOD, integrated optical density.

**Fig 12:**
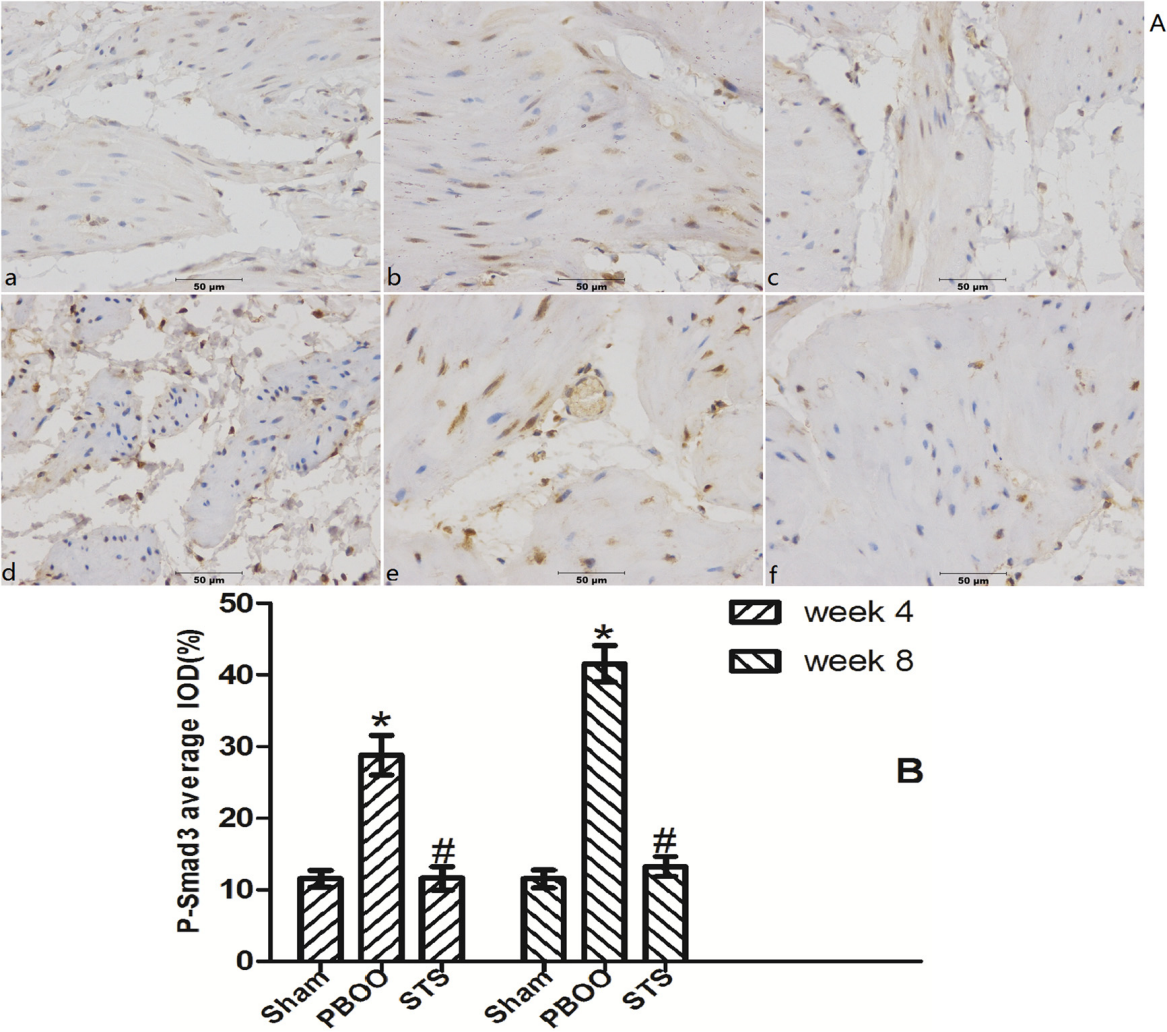
STS treatment suppresses expression of phosphorylated Smad3 in the PBOO model. (A) Representative phosphorylated Smad3-immunostained rat bladder sections. (a) Sham group at 4 weeks, (b) week 4 after PBOO, (c) week 4 after PBOO+STS treatment, (d) week 8 after Sham group, (e) week 8 after PBOO, (f) week 8 after PBOO+STS treatment. Expression of phosphorylated Smad3 was significantly higher in PBOO rats at weeks 4 and 8 compared with the Sham group (*P*<0.05, n = 8). STS treatment induced decreased expression of phosphorylated Smad3 (*P*<0.05, n = 8). (B) Densitometric analyses performed from 8 independent experiments. Each bar represents the mean±SD of 8 rats. *P<0.05 *vs*. sham group (n = 8); ^#^ *P*<0.05 *vs*. PBOO group. Original magnifications, ×400. IOD, integrated optical density.

We assessed the effects of STS on TGF-β1 (*Tgfb 1*) and *Smad2/3* mRNA expression using real-time PCR. STS inhibited the expression of (*Tgfb 1* mRNA but had no effect on *Smad2* and *Smad3* mRNA expression in PBOO rat bladder at weeks 4 and 8 (Fig 8). Western blot analysis revealed that STS treatment reduced the expression of TGF-β1 protein and phosphorylated Smad2/3, but not total Smad2/3 expression in PBOO rats at weeks 4 and 8 (*P*<0.05, n = 8, Fig 9). The immunohitochemistry experiments revealed similar results (Figs 10, 11 and 12).

## Discussion

In the present study, it was observed that bladder tissue fibrosis in the PBOO model was significantly reduced at weeks 4 and 8 by intraperitoneal injection of STS a traditional herbal medicine, indicating that STS has therapeutic effects in the amelioration of bladder wall fibrosis secondary to PBOO.

Chronic obstruction of the bladder outlet due to in benign prostatic hypertrophy (BPH) leads to a number of cellular and structural alterations in the bladder wall that ultimately impair the ability of the bladder to empty or fill completely[19]. Various conditions involving neurological or outlet obstruction could give rise to the accumulation of connective and fibrotic tissues in the bladder wall [20], which in turn, cause bladder dysfunction, and can ultimately result in renal failure. The reduced bladder compliance consequent to bladder outlet obstruction results from the dysmorphogenesis, marked by myofibroblast hyperactivity and excessive ECM deposition. Excessive accumulation of collagen fibers occurs in the bladder walls of low-compliance bladders, where the total collagen has been reported to be increased by 10%, and in particular, collagen III fiber type increased by about 49% [21]. In the present study, expression of the collagen fibers in bladder wall was significantly increased after PBOO (*P*<0.05) when compared to the Sham group. There was a time-dependent 2-fold increase in accumulation of collagen fiber in PBOO operation without STS treatment group at week 4 when compared with week 2. On the contrary, collagen fibril expression in S PBOO operation with STS treatment group rats was significantly lower than that in the PBOO operation without STS group (*P*<0.05, Fig 4), indicating that STS inhibited the collagen expression secondary to PBOO. It may be inferred that STS may aid in improving bladder compliance, and thus enhance bladder function after PBOO. Among various factors, TGF-β1 is perhaps the key cytokine that plays a central role in the progression of fibrosis. Recent reports have elucidated the TGF-β1 signaling mechanism involved in the development of fibrosis in the PBOO model [5].

The active form of TGF-β1 binds to a TGF-β-specific receptor, forming an activated complex [22]. The activated TGF-β receptor complex initiates intracellular signaling by phosphorylating specific downstream proteins known as R-Smads (Smad2 and Smad3), which in turn form complexes with the common Smad (Smad4). The activated Smad complexes get translocated from the cytoplasm into the nucleus, where the activated Smads regulate the transcription of the target genes, such as those for collagen I (*COL1A1*) and collagen III (*COL3A1*) [23]. The reduced bladder compliance consequent to bladder outlet obstruction results from the dysmorphogenesis, marked by myofibroblast hyperactivity and excessive ECM deposition. In a unilateral ureteral obstruction model, a dramatic decrease in mRNA expression of TGF-beta 1 and type I collagen was demonstrated in antisense-transfected obstructed kidneys. Further, the interstitial fibrotic area of the obstructed kidneys exposed to antisense was significantly lower than that of both untreated obstructed kidneys and kidneys treated with scrambled antisense [24]. However, its role in the progress of the bladder fibrosis subsequent to PBOO remains a major topic of debate. Using a PBOO model, TGF-β1 protein expression was found to be decreased during bladder compensation, while an elevated TGF-β1 expression was reported during the decompensation stage [25]. In our study, we found that TGF-β1 expression was elevated at 4 weeks and 8 weeks, based on which it can inferred that the bladder has gradually entered the stage of decompensation at 4 weeks. However, some reports suggest that the bladder fibrosis developed in rats, 14 days after obstruction [26, 27]. In another study, higher values of TGF-β1 were observed in a rat PBOO model, and additionally, treatment with amitriptyline further aggravated the fibrotic process after infravesical obstruction [28]. Taking a cue from these studies, we assessed TGF-β1 expression in PBOO at weeks 4 and 8 using RT-PCR and western blotting. We found that the expression of TGF-β1 mRNA and protein in the PBOO operation without STS treatment group was significantly higher than that in the sham operation group (*P*<0.05, Fig 9). TGF-β recruits fibroblasts from the surrounding tissues, and promotes their proliferation and differentiation, thereby stimulating the synthesis of ECM, for example, connective tissues and collagen fibrils [29]. In our study, STS markedly inhibited phosphorylated Smad2/3 protein levels without altering Smad2/3 expression, suggesting that it ameliorates bladder fibrosis secondary toPBOO by blocking the TGF-β1-mediated signaling pathway in a positive feedback loop.

STS has been used to treat cardiovascular disease and various tumors [30]. The potential effects of STS treatment on the progression of fibrosis in different organs or tissues have garnered much research interest recently [31–35]. We observed that α-SMA expression was significantly increased in the PBOO operation without STS treatment group. α-SMA expression is considered specific for myoblasts that could be derived from epithelial-mesenchymal transition and such cells can induce ECM deposition[29]. In the present study, STS treatment effectively inhibited α-SMA expression and TGF-β1/Smad signaling pathway activation. It can postulated from the findings of this study that STS inhibits the expression of TGF-β1 mRNA leading to decreased activity of downstream signaling pathways. STS has also been reported to inhibit renal interstitial fibroblasts differentiation via the TGF-β1/Smad signaling pathway leading to a reduction in collagen I and collagen III deposition[31]. In other words, STS could have a role as a potential therapeutic modality for attenuating ECM synthesis in PBOO, possibly by modulating TGF-β/Smad signaling.

## Conclusions

Our findings indicate that STS can delay the progression of bladder fibrosis in PBOO rats by inhibition of the TGF-β/Smad signaling cascade. The present results support the premise that STS may be a potential therapeutic choice for preventing bladder fibrosis secondary to PBOO.
